# Transcriptomic analysis of peritoneal cells in a mouse model of sepsis: confirmatory and novel results in early and late sepsis

**DOI:** 10.1186/1471-2164-13-509

**Published:** 2012-09-25

**Authors:** Minny Bhatty, Ruping Fan, William M Muir, Stephen B Pruett, Bindu Nanduri

**Affiliations:** 1 Department of Basic Sciences, College of Veterinary Medicine, Mississippi State University, Mississippi State, MS 39762, USA; 2 Department of Cellular Biology and Anatomy, Louisiana State University Health Sciences Center, Shreveport, LA 71130, USA; 3 Department of Animal Sciences, Purdue University, West Lafayette, IN 47907, USA

**Keywords:** Intra-abdominal sepsis, Microarray, Peritoneal leukocytes

## Abstract

**Background:**

The events leading to sepsis start with an invasive infection of a primary organ of the body followed by an overwhelming systemic response. Intra-abdominal infections are the second most common cause of sepsis. Peritoneal fluid is the primary site of infection in these cases. A microarray-based approach was used to study the temporal changes in cells from the peritoneal cavity of septic mice and to identify potential biomarkers and therapeutic targets for this subset of sepsis patients.

**Results:**

We conducted microarray analysis of the peritoneal cells of mice infected with a non-pathogenic strain of *Escherichia coli*. Differentially expressed genes were identified at two early (1 h, 2 h) and one late time point (18 h). A multiplexed bead array analysis was used to confirm protein expression for several cytokines which showed differential expression at different time points based on the microarray data. Gene Ontology based hypothesis testing identified a positive bias of differentially expressed genes associated with cellular development and cell death at 2 h and 18 h respectively. Most differentially expressed genes common to all 3 time points had an immune response related function, consistent with the observation that a few bacteria are still present at 18 h.

**Conclusions:**

Transcriptional regulators like PLAGL2, EBF1, TCF7, KLF10 and SBNO2, previously not described in sepsis, are differentially expressed at early and late time points. Expression pattern for key biomarkers in this study is similar to that reported in human sepsis, indicating the suitability of this model for future studies of sepsis, and the observed differences in gene expression suggest species differences or differences in the response of blood leukocytes and peritoneal leukocytes.

## Background

The hallmark of sepsis is a systemic overwhelming inflammatory response induced by an infectious agent. Despite extensive research, sepsis still remains a leading cause of death in the United States [1]. Multiple immunological molecules and pathways with redundant roles contribute to the outcome of sepsis. Microarray based transcription profiling for understanding the pathophysiology of sepsis has predominantly focused on the blood profiles from sepsis patients or animal models [2-4], with few reports on the primary organs in which the infection originated, such as lung [5] and brain [4].

Sepsis caused by intra-abdominal infections arising from a ruptured appendix, penetrating trauma and peritonitis accounts for about 20% of all sepsis cases [6], with a mortality rate of 25-35% [7]. Although sepsis is a systemic infection, studies indicate that the inflammatory response is variegated amongst different organs and peripheral blood [8-10]. This variation can be attributed to the nature of the insult or infection, and the cellular composition and the microenvironment of each organ system [11]. Several different studies have provided evidence of differential expression of cytokines and chemokines in different organs or blood in animal models of sepsis [9-13]. A study in a mouse endotoxemia model showed that the mRNA levels of IL-1β, TNF-α and MIP-2 are significantly different in neutrophils derived from lungs as compared to those from peripheral blood [12]. Similar differences in IL-1β levels in lung and blood have been reported in immunohistochemistry studies [13]. Organ specific expression of CXC chemokines (MIP-2 and KC) and a CC chemokine (RANTES) has been shown in a mouse model of peritonitis and endotoxemia [9,10]. In addition to differences in the cytokine and chemokine expression, there is evidence that the signaling and the receptors leading to the inflammatory response also vary depending upon the anatomical location of the leukocytes involved. This is exemplified by a study that showed that LPS induced NF-κB activation in liver is mediated through TNF-α and IL-1 receptor dependent pathways while in the lungs it is largely independent of these receptors[14]. Tolerance to endotoxin in a mouse model has also been shown to be compartmentalized depending upon the cell type in a given organ [14]. Therefore, it is important to evaluate the inflammatory responses at the primary focus of the infection which leads to sepsis in order to understand the progression of sepsis from the primary location of infection to a severe systemic condition and then to resolution, as the inducing microbes are cleared.

To date, genome wide transcriptional changes in cells isolated from the peritoneal fluid, the primary site of infection in intra-abdominal sepsis have not been reported. Here, using microarrays, we describe temporal (early and late) changes in inflammatory responses in cells from the peritoneal fluid of mice infected with a non-pathogenic strain of *Escherichia coli*. The mouse model used in this study involves the intraperitoneal administration of a non-pathogenic strain of *E. coli*. This is designed to represent the patient population in which sepsis is caused by microbial contamination of the peritoneal cavity due to conditions that compromise the normal mucosal barrier. The infections often start as polymicrobial but in more than half of the cases, *E. coli* is typically the only type of bacterium isolated [15,16]. This model of sepsis causes time-dependent changes that are consistent within treatment groups [17,18], whereas cecal ligation puncture and the fecal pellet models exhibit considerable variation within groups at early time points and thus are not well suited for assessment of time dependent changes [17,18].

## Results

### Animal model, bacterial and differential cell counts in peritoneal fluid

**Figure 1:**
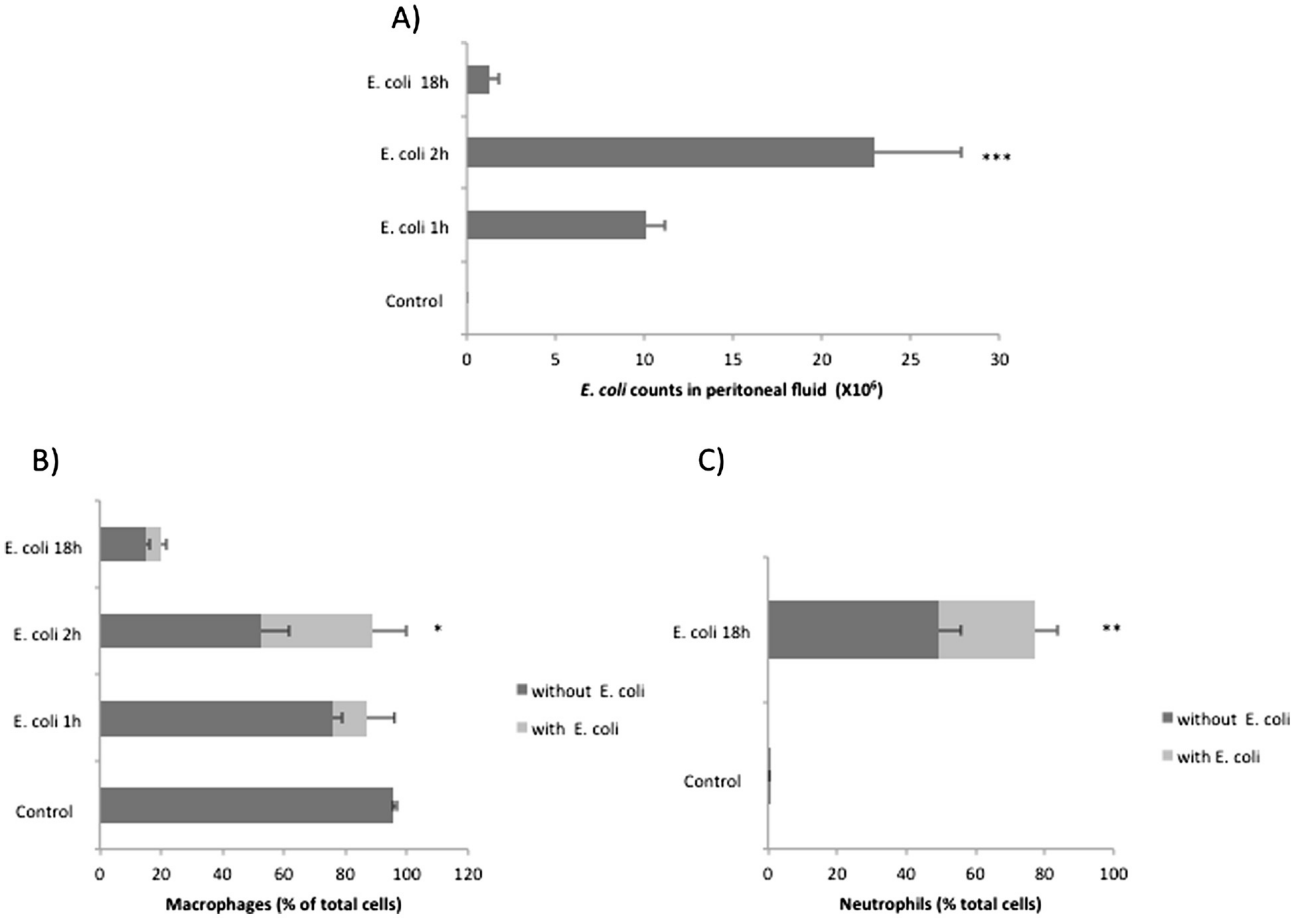
**Bacterial and cell counts in mouse peritoneal fluid at early and late time points. A**) *E. coli* counts in the peritoneal fluid of control (n = 5) and infected mice (n = 5) 1, 2 and 18 h after infection (**B**) Percentage of macrophages in peritoneal fluid of control (n = 9) and *E. coli* infected mice (n = 5) 1, 2 and 18 h post infection (**C**) Percentage of neutrophils in peritoneal fluid of control (n = 5) and *E. coli* infected mice (n = 5) 18 h post infection. The *E. coli* treated group was infected with 2 × 10^8^ cells of *E. coli* intraperitoneally. The control group received equivalent amount of PBS. The error bars indicate the standard error of mean. ***P < 0.01, *P < 0.05.

The mouse model of intra-abdominal sepsis used here is based on our previous study [19], which indicates that 18 h is about the latest time point for sampling, without the risk of animal mortality or morbidity. Levels of pro-inflammatory cytokines (TNF-α, IFN-γ and GM-CSF) are elevated as early as 1 h while most other cytokines peak within 2 h of infection [19,20]. Therefore, we chose two early (1 h, 2 h) and one late (18 h) time point in this study. The peritoneal fluid bacterial counts were highest at 2 h (Figure 1A), consistent with our previous findings [19]. By 18 h most of the bacteria were cleared (Figure 1A), which should allow the resolution of inflammation. The survival study also indicates that most of the mice survive at this dose of infection (Additional file 1: Figure S1). At 1 h and 2 h > 85% of the cells in the peritoneal fluid are macrophages (Figure 1B), and at 18 h they are predominantly neutrophils (~80%) (Figure 1C). The percentage of macrophages that contain 3 or more *E. coli* cells increases at 2 h as compared to 1 h indicating increased uptake of bacteria. Thus, the changes in gene expression noted in this study between early and late time points undoubtedly reflect both changes in gene expression and changes in the predominant cell type. However, this change in cell types represents the natural response to sepsis and the beginning of the process of resolution and the results reflect overall gene expression in peritoneal cells during this time period. Therefore, we believe that these results are useful, even though it cannot be determined with certainty which type(s) of cells express the genes detected.

### Microarray analysis

**Figure 2:**
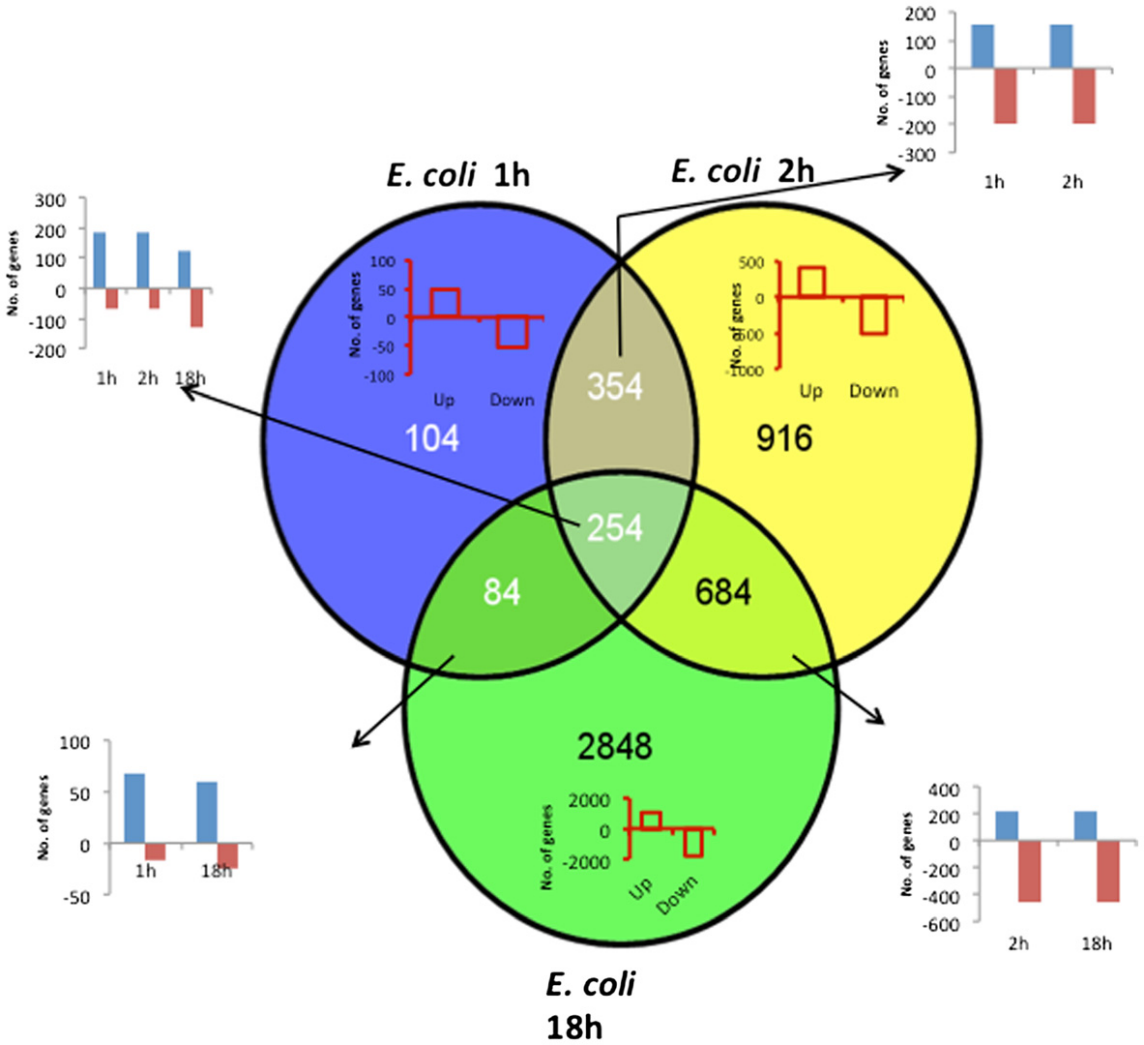
**Distribution of significant differentially expressed genes at early and late time points.** Venn diagram showing the distribution of significant differentially expressed (DE) genes at different time points.

We used the Affymetrix 430 2.0 mouse microarray platform to evaluate the early (1 h, 2 h) and late (18 h) changes in the transcriptional profiles of peritoneal cells from *E. coli* infected and control mice. The distribution of the 5244 differentially expressed (DE) genes shared among the different time points and those unique to each time point are shown as a Venn diagram in Figure 2. A majority of the DE genes were unique to 18 h, while most of the DE genes at 1 h were shared with other time points, although in some cases the direction of change is different.

### Analysis of gene expression at early and late time points

**Figure 3:**
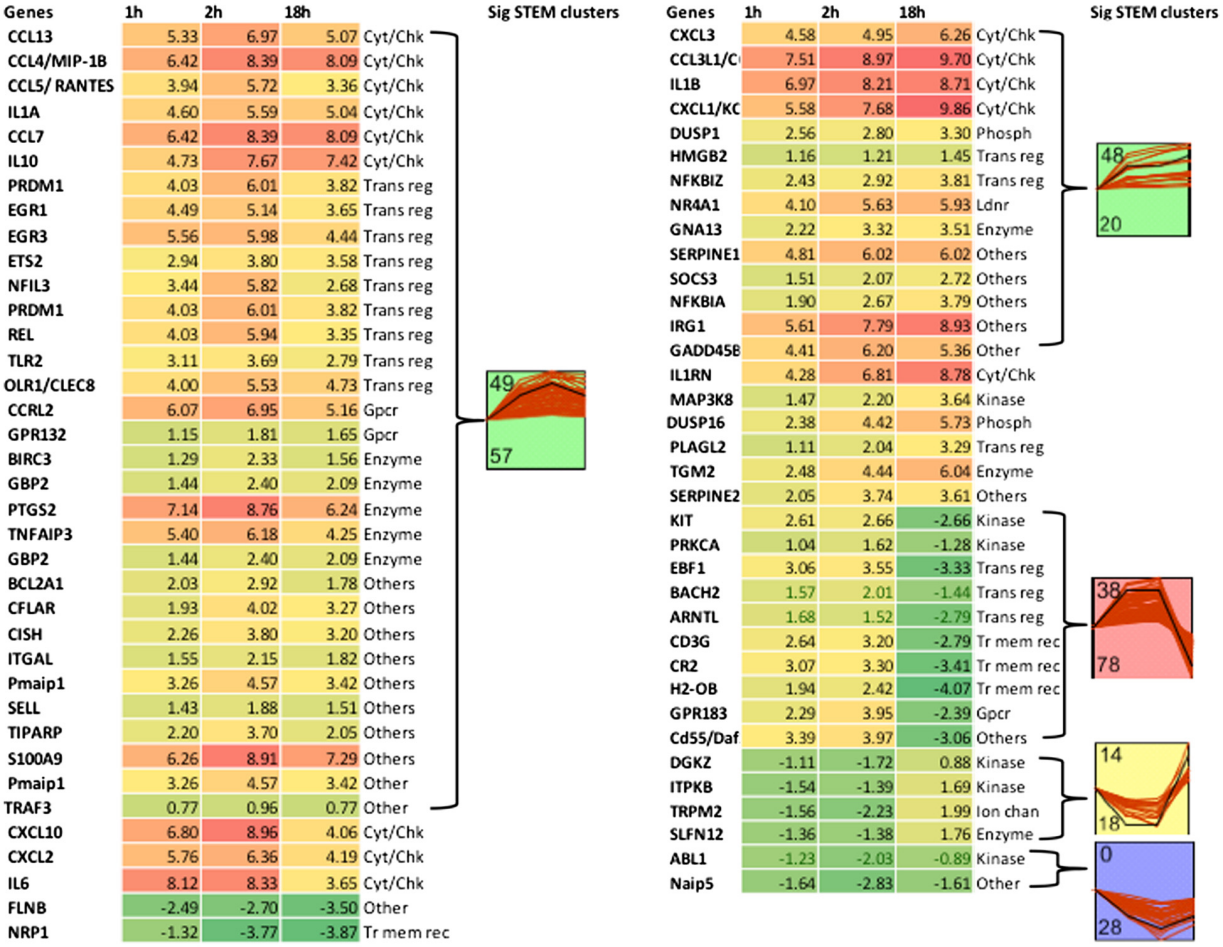
**Expression profiles of differentially expressed immune response genes common to early and late time points.** Heat map of DE immune response related genes common to 1, 2 h and 18 h time points and significant clusters identified by Short Time Expression Miner (STEM) clustering software. Within each cluster, the number on top represents the profile id and the number at the bottom represents the number of genes with similar profile patterns shown in the same colour. Sig: significant; cyt/chk: cytokine/ chemokine; trans reg: transcription regulator; Gpcr: G-protein coupled receptor; Tr mem rec: Transmembrane receptor; Ldnr: Ligand dependent nuclear receptor; Phosp: Phosphatase.

Differences in the transcription profiles between early and late time points, to some extent, will reflect the shift from macrophages to neutrophils. For example, CD86, a co-stimulatory molecule present on macrophages and dendritic cells, implicated in early phases of the immune response, [21,22] was significantly up-regulated at 2 h (DE 2.73). Expression of CD177 or NB1 a neutrophil specific antigen [23] increased only at 18 h (DE 7.40). The gene designated H2-OB is the mouse ortholog of HLA-DOB, which forms the β chain in the MHC II molecules expressed on macrophages, dendritic cells and B cells. Expression of this gene was elevated at the two early time points and significantly decreased at 18 h (Figure 3). Expression of CR2 (complement receptor 2), PRKCA (protein kinase C alpha), which are expressed by macrophages and are important for complement activation and neutrophil recruitment [24-26], had a similar pattern of expression (Figure 3).

To confirm that the chosen time points describe molecular events at the early and late stages in sepsis, we looked for expression of markers specific to these stages of sepsis. Adrenomedullin is a member of the calcitonin peptide superfamily, and is a reliable early prognostic marker of sepsis in humans [27,28], with a prognostic accuracy similar to the APACHEII score and procalcitonin expression [29,30]. Besides being a potent vasodilating agent, adrenomedullin is an immune-modulating and antibacterial mediator and a potential therapeutic target for sepsis [31]. In abdominal sepsis, adrenomedullin stabilizes the gut barrier function [32]. In our mouse model of sepsis, adrenomedullin was significantly up-regulated at the early time points (DE 3.95 and 5.03 at 1 and 2 h, respectively) while it was down-regulated at 18 h (DE −1.95) which corresponds with the recovery from sepsis. Other cell surface markers like CD40 [33] and CD69 [34] whose expression increases in severe sepsis and decreases with the resolution of sepsis were significantly up- regulated at early time points, and decreased at 18 h (data not shown).

### DE genes unique to time points

**Figure 4:**
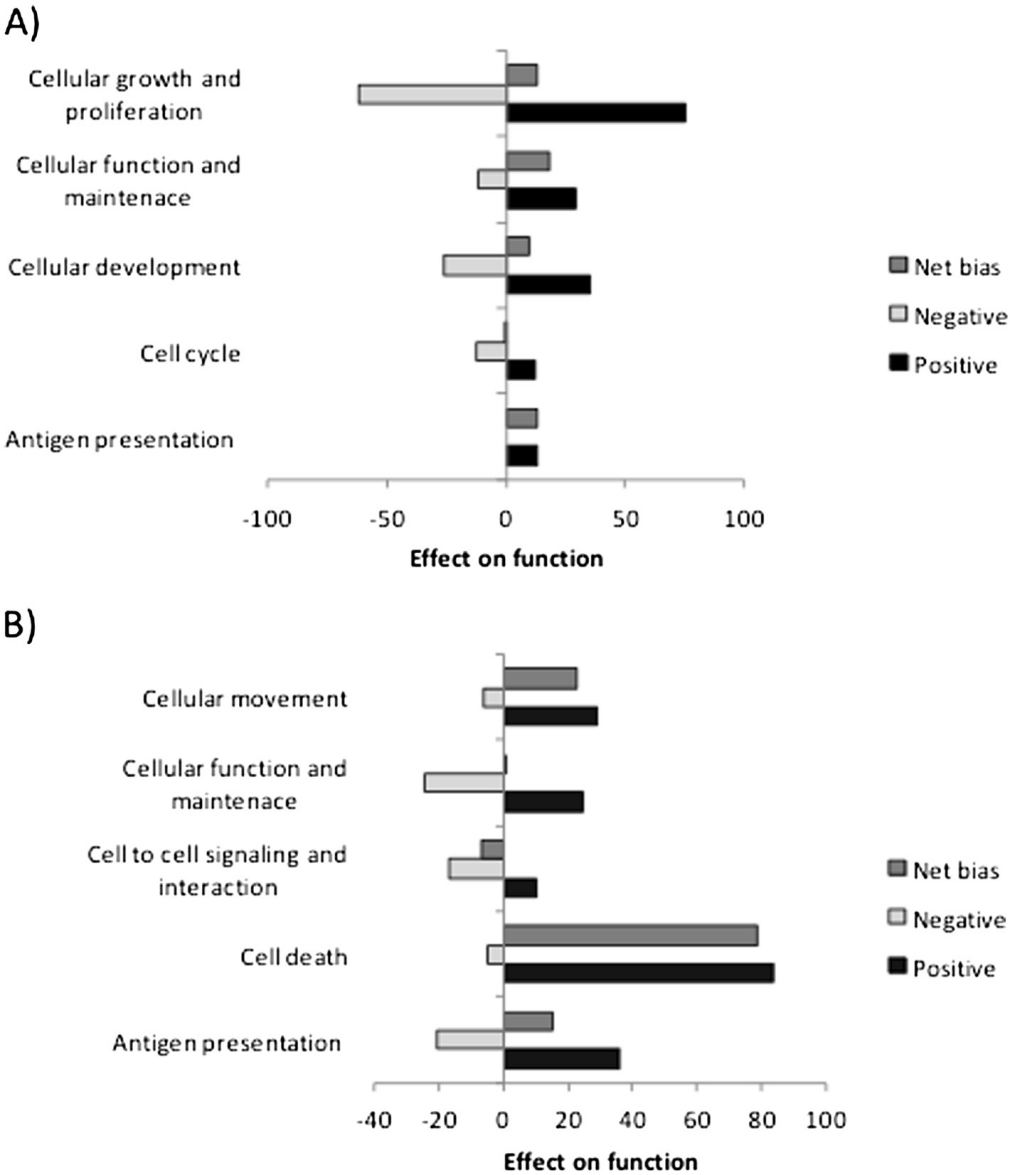
**Relative bias of functions associated with genes unique to 2 h and 18 h.** The positive, negative and net bias of the gene expression on the top functions associated with significant DE genes unique to 2 h (**A**) and 18 h (**B**) evaluated by using *GoModeler* workflow.

Gene expression that is unique to early or late time points is indicative of specific host responses at these stages of infection. The top 5 functions and canonical pathways represented by DE genes unique to early and late time points identified by Ingenuity pathway analysis (IPA) are shown in Table 1. The overall effect of the increased/decreased gene expression on the significant molecular functions (Table 1) at 2 h and 18 h was assessed by *GOModeler *[35] workflow. *GOModeler* enables Gene Ontology based hypothesis-driven interrogation of high throughput data. Since the total number of DE genes (Figure 2) and thus the DE genes associated with each molecular function (Table 1) at 1 h was too limited for meaningful interpretation by *GOModeler*, they were excluded from analysis. The net effect of DE at 2 h is positive for “antigen presentation”, “cellular development”, “cellular function and maintenance”, and “cellular growth and proliferation” (Figure 4A). Some of the top canonical pathways at 2 h are “dendritic cell maturation”, “TREM signaling” and “Role of JAK family kinase in IL-6 cytokine signaling” (Table 1). Taken together these results indicate the initiation of an inflammatory response at 2 h in response to the bacteria. The up-regulation of genes associated with dendritic cell maturation could indicate the presence of maturing dendritic cells in peritoneal fluid, or perhaps more likely, the activation of macrophages (the predominant cell type in the peritoneal cavity at 2 h), which express many of the same markers as dendritic cells.

**Table 1:** Top five functions and canonical pathways unique to 1 h, 2 h and 18 h obtained by Ingenuity pathway analysis

	**Top functions**	**Top Canonical pathways**
Unique to 1 h	Cell cycle	CD 40 signaling
	Cell to cell signaling and interaction	p53 signaling
	Cellular assembly and organization	HMGB1 signaling
	DNA replication, recombination and repair	Cholecystokinin/Gastrin- mediated signaling
	Carbohydrate metabolism	Type I diabetes mellitus signaling
Unique to 2 h	Cellular development	Dendritic cell maturation
	Cellular function and maintenance	Type I diabetes mellitus signaling
	Cell cycle	Role of JAK family kinase in IL-6 type cytokine signaling
	Cellular growth and proliferation	TREM1 signaling
	Antigen presentation	Altered T cell and B cell signaling in rheumatoid arthritis
Unique to 18 h	Cellular movement	NF-κB activation by viruses
	Cell to cell signaling and interaction	Glioma signaling
	Cellular function and maintenance	B cell receptor signaling
	Cell death	LPS-stimulated MAPK signaling
	Antigen presentation	FGF signaling

**Figure 5:**
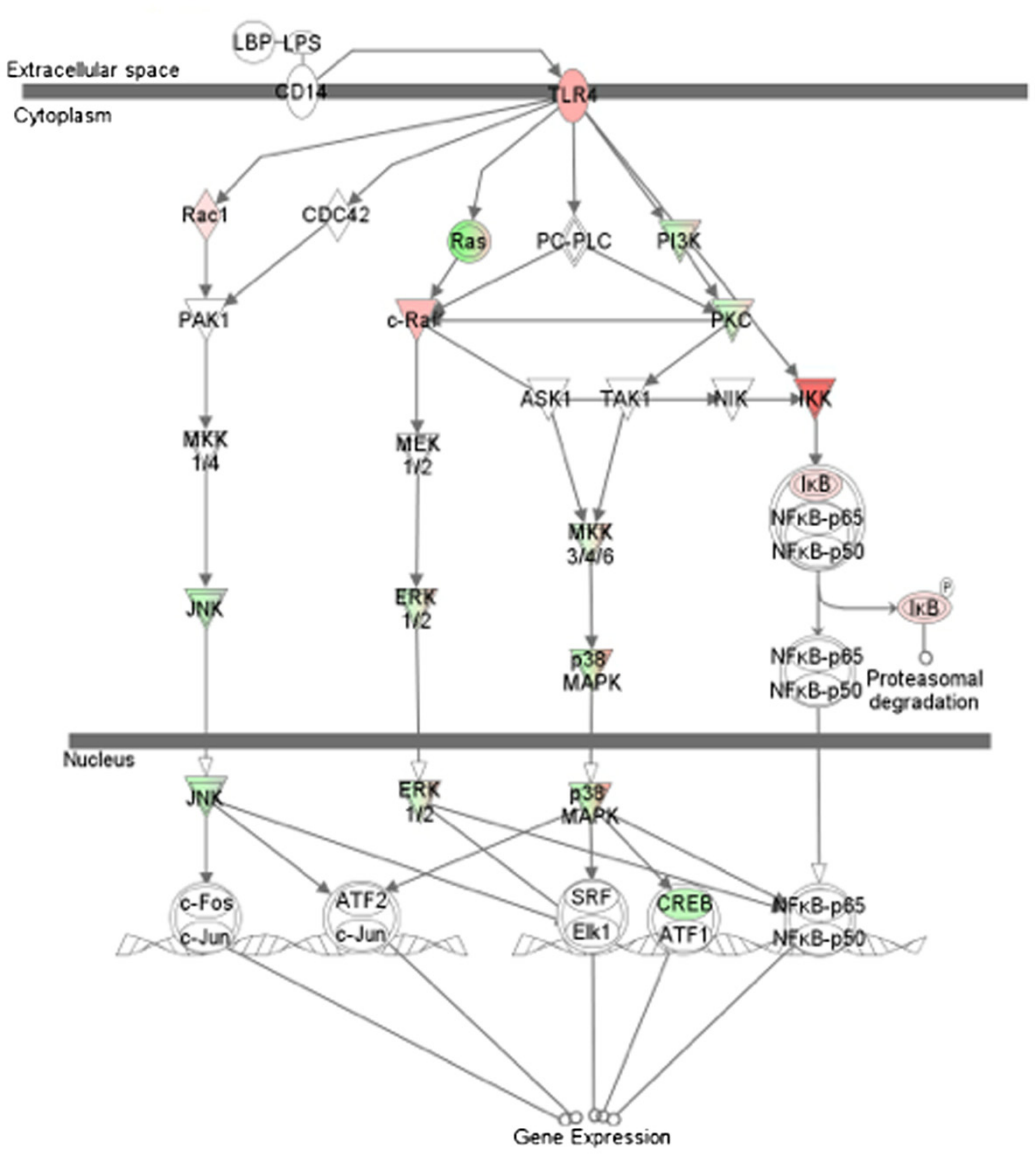
**LPS stimulated MAPK pathway representing gene expression changes unique to 18 h.** Canonical LPS stimulated MAPK pathway (shown in grey) from Ingenuity pathway analysis. Significant gene expression changes unique to 18 h in this study are highlighted in red (up-regulated) and green (down-regulated).

A significant number of DE genes (275) uniquely expressed at 18 h are associated with cell death. Most of these include apoptotic genes involved in the apoptosis of immune cells. *GOModeler* analysis shows a net positive bias for “cell death”. Pro- apoptotic genes like BCL10, BCL2L11 and FAS are up-regulated at 18 h. DE genes associated with “cell to cell signaling and interaction” had a net negative bias (Figure 4B). LPS mediated MAPK signaling (Figure 5) identified at 18 h by IPA shows that genes associated with induction of cytokine mediated innate immune response are down-regulated while inhibitors of NF-κB are up-regulated. The overall pattern of gene expression at 18 h indicates an initiation of resolution of the acute inflammatory response observed at the early time points.

### Analysis of DE genes common to all time points

DE genes (254) common to all time points were subjected to hierarchical clustering and Short Time series Expression Miner (STEM) analysis. The dendrogram associated with the hierarchical clustering revealed that 1 h and 2 h groups were similar to each other compared to 18 h (data not shown). STEM based significant clusters show similar trends of expression (up- or down-regulated) at the early time points.

The top functions of the DE genes common to all time points are related to immune or inflammatory response. Figure 3 represents the temporal trend of the transcriptional changes of the immune response related genes and the corresponding clusters which were statistically significant in STEM analysis. A majority of these genes code for cytokines, transcription regulators, kinases, and transmembrane receptors. Key pro- and anti- inflammatory mediators like IL-1α, MIP-1β, RANTES, CXCL-1, IL-6 and IL-10, implicated to play a role in sepsis were up-regulated at both the early and late time points (Figure 3). This trend was not surprising, as it is recognized that both pro- and anti- inflammatory responses are regulated simultaneously from early stages of sepsis. [36]. Our earlier findings [19,20] and current results demonstrate that a small number of viable *E. coli* are present in peritoneal lavage fluid at 18 h. Therefore, it is not surprising that a number of molecules related to immunity and inflammation are still expressed at 18 h.

Two profiles (id 38 with 78 genes, and 14 with 18 genes) have opposing trends at early and late time points (Figure 3). Some of the immune response related genes with opposing trends of expression at early (up-regulated) and late (down-regulated) time points includes, PRKCA (protein kinase C alpha), CR2 (complement component receptor 2), and CD3G involved in T cell co-stimulation. PRKCA is important for FcγR mediated phagocytosis by macrophages [37], neutrophil chemotaxis [38], and respiratory burst in neutrophils [26]. It is interesting to note that most of the immune response genes belonged to cluster 49, a significant STEM cluster that represents the trend where most of the genes were up-regulated at 2 h and their expression began to decrease by 18 h. This pattern indicates that by 18 h the peritoneal cells exhibit the initiation of resolution of infection.

In addition to transcriptional regulators like Rel (NFκBIZ), whose role in inflammation and sepsis is well established, the list of DE immune response related genes common to all time points also has two potentially novel transcription regulators (PLAGL2 and EBF1), not previously known to be involved in sepsis (Table 2). The PLAGL2 gene product induces the expression of a pro-apoptotic protein Nip-3 which causes cellular apoptosis [39]. Compared to the early time points, the expression of PLAGL2 was higher at 18 h. Expression of the EBF1 gene (early B cell factor 1), which decreases the differentiation of mouse pro B lymphocytes [40], was up-regulated at early time points and down-regulated at 18 h. Expanding the transcription regulators analysis to all DE genes with ≤ −2 or ≥ 2-fold expression change, and an immune response related function, we identified additional potentially novel transcription regulators in sepsis. In all, 20 potentially novel transcription regulators involved in sepsis were identified (Table 2).

**Table 2:** Differential expression of potentially novel transcriptional regulators involved in sepsis in the mouse peritoneal cells at different time points

**Group**	**Transcriptional regulators (DE values)**
Common to 1, 2 and 18 h	PLAGL2 (1.11, 2.04, 3.29), EBF1 (3.06, 3.55, -3.33)
Common to 1 and 2 h	ELF1 (1.32, 1.69)
Common to 2 and 18 h	AFF1 (2.55, 2.86), SKIL (3.76, 3.82)
	CBFA2T3 (−2.16, -2.34), HHEX (−2.71, -2.08)
Unique to 2 h	HIVEP2 (3.77), TCF7 (3.70), JARID2 (2.74), ZEB (2.11), KLF (−1.47)
	LYL1 (−2.22)
Unique to 18 h	EPAS1 (−5.71), BCL11A (−3.40), TAL1 (−2.72), CDKN2C (−2.47), MED7 (4.55),
	SBNO2(3.28), ZBTB7B (2.43)

DE: Differential expression.

**Figure 6:**
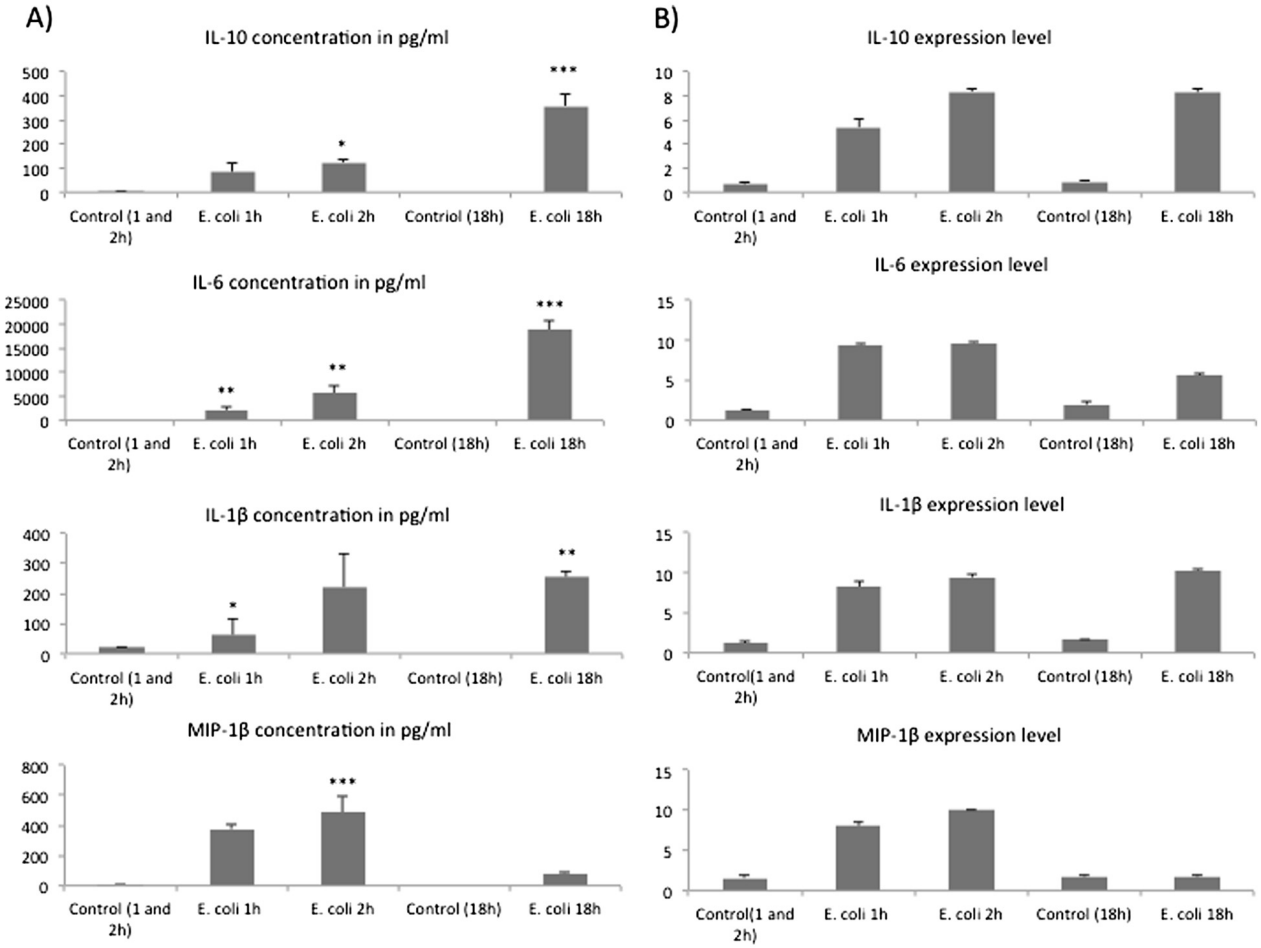
**Peritoneal fluid cytokine concentrations and corresponding gene expression determined by microarrays.A**) Cytokine concentrations measured by bioplex (n = 5) from the peritoneal fluid of control and naive and infected mice after 1, 2 and 18 h of intraperitoneal inoculation of 2X10^8^ cells of *E. coli* ***P < 0.001, **P < 0.01, *P < 0.05. (**B**) Corresponding gene expression determined by microarrays in significant differentially expressed genes.

To increase confidence in the biological interpretation of our microarray data, we determined the concentrations of three cytokines and a chemokine (IL-10, IL-6, IL-1β and MIP-1β), using a multiplex kit. The overall trends in cytokine concentrations were consistent with those observed with microarray except for IL-6. IL-6 protein concentration was highest at 18 h while the gene expression was maximal at 2 h (Figure 6). However, expression was increased compared to untreated controls at 2 and 18 h.

## Discussion

Sepsis is the 10^th^ leading cause of death in the United States [41], and the mortality rate has not changed substantially in many years, in spite of the use a wide range of experimental therapeutics. Biological based therapies that work well in rodent models when given before challenge have typically not worked in human sepsis patients. This could be due to ineffectiveness of administration of these drugs after sepsis is established or the complex biological response with many partially redundant mediators, causing inhibition of a single molecule to be ineffective. A better understanding of the full range of gene expression changes associated with sepsis will be necessary to understand these issues, and this was the purpose of the present study.

Sepsis is a result of a complex systemic dysregulation of inflammation that results when the host is unable to contain an infection that usually starts in a primary organ. The inflammatory response to sepsis involves two distinct phases which are not always mutually exclusive. The initial acute phase is the systemic inflammatory response, which is closely followed by the compensatory anti-inflammatory response [17]. The dysregulation of the immune response observed in sepsis is a combination of the local immune response at the primary site of infection and the systemic immune response. The site of primary infection plays a key role as it produces inflammatory molecules that modulate the systemic immune response. The cellular environment of each primary organ involved in sepsis including the immunological functional cells (e.g., the resident macrophages) is very specific also the microbial aetiology leading to the infection also differs considerably. Therefore it is expected that the local immune response in sepsis varies depending on the site of primary infection and thus it is important to evaluate the organ specific immune responses.

The abdomen is the second most common primary site of infection in sepsis. Intra-abdominal infections account for about 20% of all sepsis cases. We used a widely accepted [19,20,42-44] model of intraperitoneal inoculation of a non-pathogenic strain of *E. coli* to induce sepsis. Two early time points (1 h and 2 h) and one late time point (18 h) were used to evaluate the changes in the transcriptional profiles of cells in the peritoneal cavity of the infected animals. As described in the results, the early time points that are associated with increased bacterial counts are expected to reflect an acute inflammatory response. In our experience the sepsis model used in this study is associated with very low mortality, only one out of the ten mice in the survival study succumbed to infection (Additional file 1: Figure S1). This is expected since a non-pathogenic strain of *E. coli* was used for infection and most animals were able to fully clear the bacterial load by about 21 h post infection [19]. Thus the 18 h time point chosen in the study gives a view of the transcriptional changes involved in initiation of the resolution of infection. The coordinated response of professional phagocytes (macrophages, monocytes and neutrophils) is essential for the resolution of infection. The bacteria were recognized by the resident peritoneal macrophages because of their proximity to the site of infection. Thus, most of the cells in the peritoneal fluid at the early time points were macrophages (Figure 1B). The significantly increased expression of markers associated with macrophages was also reflected in the microarray data at the early time points. The cytokines released by the macrophages recruit the neutrophils from the blood stream to the site of infection. So as the infection progresses, the cell population in peritoneal fluid predominantly consists of neutrophils (Figure 1C). This shift was also reflected in the microarray data at 18 h. Most of the cells at the early time points were macrophages while at 18 h neutrophils were the predominant cell type in the peritoneal fluid (Figure 1B, 1C). The 18 h time point in our mouse model is expected to correspond to the initiation of the resolution of infection. The significant DE genes at this time point were found to be associated with cell death of immune cells. The apoptosis of neutrophils is critical for the resolution of infection as it ends the sustained neutrophil recruitment and the phagoctytic clearance of the apoptotic neutrophils also re-programs the macrophages to an anti-inflammatory phenotype [45].

We compared our results with a recent meta-analysis of 12 studies that evaluated the blood profiles from early and late stages of sepsis from human subjects [3]. In this meta analysis, there was no clear transition between pro- and anti-inflammatory phases at early or late stages. The up-regulation of TNF-α, IL-1α, IL-1β, CXCL-10, SOCS3, IL-10 at 1 and 2 h in the present study was similar to that observed in blood profiles of sepsis patients at early stages [11,46]. Expression of these genes peaked at 2 h and was still up-regulated at 18 h in this study. Decreased expression of CD69, CD36 (DE 1.78 and −5.53 respectively at 18 h) and MHC II genes (H2-OB, Figure 3) in peritoneal cell profiles is consistent with the blood profiles of sepsis patients at late stages of sepsis [47]. However, there are some differences in expression of some genes at 18 h, as would be expected due to differences in inflammatory responses in different anatomical locations [8-10]. For example, TNF-α levels in peritoneal cells is elevated at 18 h in our study (DE 5.57), but meta-analysis identified a decreased expression of TNF-α in blood profiles [36,47]. The CCR2 and S100A11 genes are up-regulated in early stages of sepsis in blood [48] but down-regulated in the peritoneal cells in our sepsis model. Despite a number of similarities, gene expression in peritoneal cells differs from the patterns in blood, suggesting that cell type and anatomical location as well as species differences influence the specific characteristics of the local inflammatory response. Nevertheless, there were many similiarities between the early and late responses to sepsis in humans and mice.

An important finding of this study is the identification of 20 transcription regulators among the DE genes, whose role in sepsis was not previously known. Some of the transcription regulators expressed uniquely at 2 and 18 h are known to be involved in T cell regulation. Transcription factor 7 (TCF-7) was up-regulated and Kruppel like factor 10 (KLF10) was down-regulated at 2 h. TCF-7 is a critical regulator of T cell specification [49] while KLF10 induces TGF-β1 expression. TGF-β1, an anti-inflammatory cytokine, also regulates T cell activation [50]. ZBTB7B, a zinc finger protein crucial for the commitment of MHCII restricted thymocytes to a CD4+ lineage [51] was up-regulated at 18 h. Strawberry notch homolgue2 (SBNO2) was also up-regulated at 18 h. SBNO2 is induced by IL-10 and contributes to its downstream anti-inflammatory effects [52]. It is not clear if the expression of T cell-related transcription regulators indicates the presence of T cells in the peritoneal cavity or if these transcription regulators are also expressed in macrophages, and have not been recognized as having a role in these cells. Lymphocytes are present in the peritoneal cavity (~5-10% of total cells, data not shown), and we have previously observed expression of genes unique to T cells (e.g., TCR), so it is possible that T cell activation is an important event in sepsis. It is also possible that a set of transcription regulators not previously known to be expressed by macrophages are expressed and are potentially important in regulating inflammation. Whether changes in expression of these regulators at 18 h is due to altered expression in macrophages or differential patterns of expression in neutrophils vs. macrophages, they are significant compared to naive mice, thus implicating them in the host response to sepsis. It is not clear if these transcription factors are activated by known or previously unrecognized signaling pathways. We report here that the best known transcription factor associated with sepsis, NF-κB, exhibits no alteration in transcript quantity. However, transcriptomics does not reveal information about activation of signaling or transcription related molecules. Our previous study demonstrated that sepsis substantially increases activation of NF-κB using a transgenic reporter mouse model [20]. This serves as a reminder that the changes in the quantity of transcripts for transcription factors may not correspond with activation of those transcription factors. All other things being equal, increased expression should yield increased activity, but activity needs to be measured for each transcription factor implicated in this study. This will be done in future studies.

## Conclusions

In conclusion, the results of this study provide an evaluation of the changes in the transcriptional profile of peritoneal cells at early and late stages of intra-abdominal sepsis. The pattern of expression of key biomarkers for sepsis in humans is similar to that observed in this study, validating the findings of this study, and suggesting the utility of this model for further studies of sepsis. We identified several novel transcription regulators which might play an important role in immune response to sepsis and could be useful diagnostic markers or therapeutic targets. Future experimental studies are needed to validate and determine the role of these transcription regulators in sepsis.

## Methods

### Mice, treatments, and procedure

We used 12–16 weeks old female C57Bl/6 × C3H F1 mice obtained through the National Cancer Institute’s Animal Program for all experiments. The mice were acclimatized for at least two weeks in filter top shoebox cages (5/cage) in a temperature (70-78°F) and humidity (40-60%) controlled environment in accordance with NIH and Louisiana State University regulations. The care and use of mice in this study were approved by the LSU Health Sciences Center Institutional animal care and use committee. Sentinel mice periodically housed in the same room as experimental animals were negative for infectious agents during the period of this study.

We divided mice into control and *E. coli* treated groups. The treated group received 2 × 10^8^ cfu of a non-pathogenic strain of *E. coli* harvested in mid-log phase, suspended in a phosphate buffered saline (PBS) solution, intraperitoneally as described [19]. The non-pathogenic isolate of *E. coli* used in the study was isolated from the colon of one of the mice in our specific pathogen free colony. The control mice were injected with an equal volume of PBS intraperitoneally. We euthanized mice 1 h, 2 h and 18 h post *E. coli* administration by inhalation of isoflurane and performed peritoneal lavage as described [53] A group of mice which received PBS was harvested at 2 h served as a common control group for 1 h and 2 h. The use of a common control group harvested at 2 h for both the early time points is unlikely to affect the interpretation of the 1 h results as the only difference expected between these two control groups would be related to circadian changes in expression of some genes. It must be considered that these differences would be quantitatively small in almost all cases after only 1 h of difference as extrapolated from results of Akhtar and colleagues, who evaluated gene expression every 4 h [54]. Peritoneal lavage fluid was also obtained from a second group of mice receiving PBS intraperitoneally after 18 h, this served as a control group for the 18 h treatment. A group of 10 mice were used for survival studies. These mice were inoculated with 2 × 10^8^ cfu of a non-pathogenic strain of *E. coli* intraperitoneally.

### Bacterial and cell counts from peritoneal fluid

An aliquot of peritoneal fluid at each time point was serially diluted in Luria Bertani agar to determine the bacterial counts after overnight incubation at 37°C. We centrifuged the remaining peritoneal fluid, and used the pellets for cell counts and RNA extraction, and the supernatant for cytokine/chemokine analysis. The total cell counts were done using Coulter Z1 particle counter (Hialeah, FL). Differential cell counts were determined by cytospin preparations. Following cytospin, the cells were stained with Wright-Giemsa stain and observed under 600 × magnification under an oil immersion lens to differentiate between the different cells types. In order to assess the phagocytosis of bacteria by the macrophages and neutrophils we used a criteria where the cells with three or more bacteria associated were referred to as cells with *E. coli*, and cells with less than three bacteria were referred to as cells without *E. coli* as described previously [19].

A criteria of 3 or more bacteria was used to account for the possibility that some of the bacteria that appeared to be intracellular might actually be on the cell surface. Although the method used here does not provide a precise quantitation of the phagocytosis but it gives a good estimation of the temporal changes in phagocytosis by the different cells types.

### Cytokine and chemokine assays

Concentrations of select cytokines and chemokines in peritoneal fluid were determined by Bio-Rad (Hercules, CA) multiplexed bead array kits following manufacturer’s protocols and with a BioPlex analyzer (Bio-Rad).

### RNA isolation, microarrays and functional analysis

We extracted RNA from the peritoneal cells of *E. coli* challenged and control mice after taking a small sample for cytospin analysis. The RNA was isolated using the TRIZol protocol (Invitrogen, Stockholm, Sweden). An Agilent Bioanalyzer was used to assess the quality of the RNA samples. Samples were selected for analysis in part by the 28S/18S ratio with a value of 1.4 or greater as the criterion for use. The microarray analysis was done as described previously [53] except that we used Mouse 430 2.0 GeneChip® microarray. The primary labeled cRNA was synthized from 7.5 μg total RNA using the Affymetrix one cycle labeling kit. We performed hybridization, staining, washing, and scanning of the Mouse 430 2.0 GeneChip® microarray according to Affymetrix protocols. Using GeneSifter (Geospiza, Perkin Elmer Inc) we normalized raw data by robust multichip algorithm (RMA). Box-whisker plots of expression values before and after normalization show that the RMA adjusted to the same median value with similar 1st and 3rd quartiles and similar second and 98th percentiles. The microarray data was submitted to NCBI GEO with accession number GSE34114.

For statistical analysis, a pooled within treatment error was computed and this was used to perform t tests for comparison at each time point i.e. at each time point the control is compared to the treatment group. To account for the false positives, the P values were adjusted for multiple comparisons using a 5% false discovery rate. We used genes that were significantly up- or down-regulated at different time points for further analysis. Differential expression for each gene represents the difference between the expression values of *E. coli* treated and the respective control.

We analyzed DE genes common to all time points by hierarchical clustering (average linkage clustering metrics and Pearson correlation for the distance) and generated heat maps with TIGR MeV (Multi Experiment Viewer) V4.3.01 software [55]. We determined statistically significant clusters in our time course experiment by using the software tool Short Time series Expression Miner (STEM version v1.3.6) [56]. To generate a common baseline for the temporal assessment with STEM, we normalized the data by adding a “0” for each gene.

We identified significant functions, canonical pathways and networks associated with the DE genes using Ingenuity pathway analysis (Ingenuity systems, Redwood City, California, USA). The net effect (positive or negative bias) of gene expression changes specific to 2 and 18 h on the top 5 functions identified by IPA was evaluated using *GoModeler*[35].

## Abbreviations

APACHE: Acute physiology and chronic health evaluation; BCL: B-cell leukemia/lymphoma; BCL2L11: BCL2 like 11; CD: Cluster of differentiation; CR: Complement receptor; CXCL: Chemokine (C-X-C) motif ligand; DE: Differentially expressed; EBF1: Early B cell factor 1; GO: Gene ontology; GM-CSF: Granulocyte macrophage-colony stimulating factor; H2-OB: Histocompatibility 2 O region beta locus; IFN: Interferon; IL: Interleukin; IPA: Ingenuity pathway analysis; JAK: Janus kinase; LPS: Lipopolysaccharide; MAPK: Mitogen activated protein kinase; MHC: Major histocompatibility complex; MIP: Macrophage inflammatory protein; NF-κB: Nuclear factor of kappa light polypeptide gene enhancer in B-cells; PRKCA: Protein kinase C alpha; RANTES: Regulated upon activation normal T-cell expressed and secreted; PLAGL: Pleiomorphic adenoma gene-like 2; SOCS: Suppressor of cytokine signaling; TREM: Triggering receptor expressed on myeloid cells; TNF: Tumor necrosis factor.

## Competing interests

All authors declare no competing interests.

## Authors’ contribution

MB analysed and interpreted the data and wrote the manuscript, RF performed the experimental procedures, WMM did the statistical analysis of microarray data, SBP designed the study and helped with the data interpretation and manuscript preparation, BN helped with data analysis, interpretation and manuscript preparation. All authors have read and approved the manuscript.

## Supplementary Material

Additional file 1: Figure S1 Survival studies in the mouse model of sepsis. A group of 10 mice were inoculated with of 2 × 10^8^ cells of *E. coli* intraperitoneally and observed for survival. Click here for file
